# Environmental drivers of wild orchid distribution: Soil properties shape habitat preferences in the Po Delta Regional Park (Italy)

**DOI:** 10.1371/journal.pone.0340676

**Published:** 2026-02-18

**Authors:** Lisa Scramoncin, Renato Gerdol, Anna Cazzavillan, Fabio Vincenzi, Lisa Brancaleoni

**Affiliations:** Department of Environmental and Prevention Sciences, University of Ferrara, Ferrara, Italy; National Cheng Kung University, TAIWAN

## Abstract

European terrestrial orchids inhabit a wide range of habitats, including forests, grasslands, wetlands and anthropogenic ecosystems. This study investigates the ecological preferences of seven wild orchid species in the Po Delta Regional Park (Northern Italy), focusing on how environmental gradients of soil properties such as moisture, salinity, pH, and nutrient availability shape species distribution and niche breadth. We conducted vegetation surveys and field measurements of soil variables across 27 sites. Species differentiation along environmental gradients was analysed through Multivariate Analysis of Variance (MANOVA), Principal Component Analysis (PCA), and Outlying Mean Index (OMI) analysis to assess niche breadth and marginality. We then compared normalized Ellenberg Indicator Values (EIVs) for Moisture, Reaction, Salinity, and Nutrients with measured soil variables [Volumetric Water Content, pH, salinity, ammonium (NH_4_^+^), nitrate (NO_3_^−^), and phosphate (PO_4_^3−^) concentrations] using Linear regression. Moisture and salinity were key drivers of orchid distribution. *Anacamptis laxiflora* and *Anacamptis palustris* were associated with wet, saline environments, whereas *Anacamptis pyramidalis* and *Anacamptis coriophora* preferred drier, nutrient-poor soils. *Anacamptis laxiflora* exhibited highest marginality and narrowest niche breadth, indicating high specialization. In contrast, *Ophrys apifera* showed lowest marginality and highest tolerance, reflecting its generalist strategy and adaptability to a broad range of soil conditions. EIV-Moisture and EIV-Salinity showed strong correlations with field measurements, whereas EIV-Nutrients and EIV-Reaction were less predictive. These findings emphasize the importance of conserving environmental heterogeneity, especially in human-dominated ecosystems, to support both generalist and specialist orchid species.

## 1 Introduction

Ecological niche is a key concept in modern ecology, concerning both the range of conditions necessary for the survival of species and all mutual interactions among species and their interaction with the biotic and abiotic environment [1]. Many factors can shape the ecological niche. Macroclimate and evolutionary and migration histories influence the distribution of species across large geographical areas [2,3]. On a regional scale, factors such as soil physical and chemical characteristics, meso- and micro-climate, light regime, habitat type, community composition and disturbance regime play a key role in determining species abundance and richness [4,5]. Global climate change, habitat loss and fragmentation, overexploitation of wild species and abandonment of traditional habitat management are just some of the factors that can negatively affect plant species, particularly those with specific ecological niches, which depend on specific conditions for their germination, growth and reproduction [6,7]. Thus, a thorough understanding of the ecological preferences, along with analyses of the major drivers that influence the diversity and distribution patterns of species, is crucial for understanding how species respond to changing environmental conditions and, therefore, for the development of effective conservation strategies for both species and their habitats [8–11].

With over 28,000 species and 763 genera, *Orchidaceae* is among the largest families of flowering plants [12]. Orchids inhabit a wide range of environments worldwide, with most species thriving in tropical regions [13]. In Europe, the Mediterranean region is one of the most important hotspots for orchid diversity, where all orchid species are terrestrial and can be found in several different habitats, including forests, grasslands, bogs, marshlands, and even anthropogenic ecosystems [14]. Despite this great diversity, many orchid species faced a drastic decline in abundance and distribution over the past few decades due to habitat loss and changes in land-use practices [15]. Therefore, several species have turned rare or endangered, and many countries have prompted their protection under national and international legislation, as well as their inclusion in Red Data Books [16]. Moreover, native orchid species show a remarkable level of ecological specialisation, as they rely on complex ecological associations with specific pollinators and mycorrhizal fungi for their survival and reproduction [17]. Consequently, these plants are considered important bioindicators of the ecological quality of ecosystems due to their high vulnerability to environmental changes [18]. All these features highlight that, in highly industrialised European countries, conservation of orchid species presents a considerable challenge, primarily due to widespread habitat degradation and the absence of suitable environmental conditions necessary for their long-term survival [19].

Different studies pointed out that orchid distribution can be closely linked to factors such as soil moisture content, nutrient availability, light regime, climatic variables and competition levels [20,21]. Although several European studies examined the relationship between terrestrial orchid diversity and ecological factors across large geographic extents [22–24], the impact of these drivers can differ among regions so that a universally consistent pattern of orchid diversity cannot be generalized across all species and geographic areas. Therefore, studies at local scale are crucial for enhancing the understanding of how species richness interacts with environmental variables, enabling effective local conservation and habitat management [25,26]. The *Orchidaceae* family is well represented in Italy’s native vascular flora, comprising 236 taxa across 27 genera with 87 endemic taxa [27]. All species are legally protected at the regional level by Regional Law No. 2 (24 January 1977), and at the national and international levels by the Habitat 92/43/EEC Directive, the Convention on the conservation of European wildlife and natural habitats (Bern Convention) and the Convention on International Trade in Endangered Species of Wild Fauna and Flora (CITES) [28]. Our case study was conducted in different semi-natural grasslands of the Po Delta (Northern Italy), which is regarded as one of the most important natural areas in Europe [29]. However, this territory has undergone substantial anthropogenic alteration due to urbanization, tourism and agricultural expansion [30]. As a matter of fact, many natural habitats, including pine forests, wet and dry meadows and grasslands, have been destroyed or converted into urban green spaces. Over time, these areas experienced an increase in non-native plant species with a high tolerance for a wide range of environmental conditions, leading to a decline in the natural populations of wild orchids, which are generally weaker competitors [31]. Furthermore, these semi-natural grasslands are subject to additional pressures such as uncontrolled herbivory and inappropriate mowing regimes. Specifically, both frequent mowing, driven by urban aesthetic policies, and the complete abandonment of traditional management practices led to the degradation of these habitats, resulting in the loss of key ecological niches and a decline in vascular plant species richness [32,33].

This study investigates the ecological preferences of seven wild orchid species, focusing on site-specific soil properties and associated vegetation assemblages. The main objectives were: (i) to measure species’ niche breadth and marginality, (ii) to identify the main environmental drivers influencing their occurrence and (iii) to verify the correlation between Ellenberg Indicator Values (EIVs) and field measurements to assess their reliability as predictors of environmental conditions. These insights are relevant for implementing appropriate management strategies for semi-natural meadows and for preventing both habitat degradation and species decline.

## 2 Materials and methods

### 2.1 Study area and species selection

**Fig 1:**
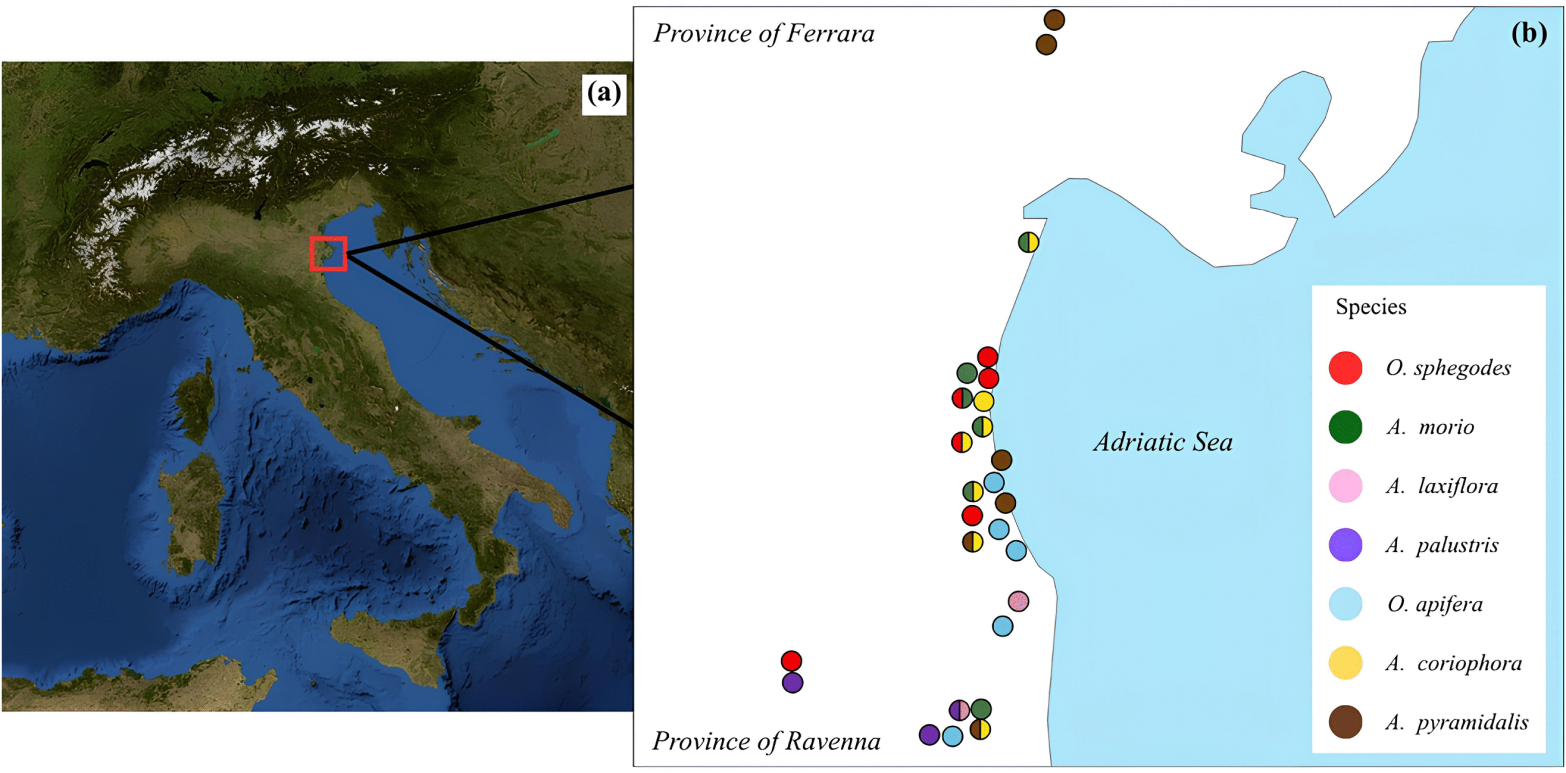
Map of the study area. The red square indicates the location of the study area in Italy (panel a). The detailed map in panel b shows the sampling sites distributed along the coastal zones of the Provinces of Ferrara and Ravenna. Basemap from the U.S. Geological Survey (USGS ImageryOnly, The National Map) – *public domain*. Sampling sites were added by the authors.

The study was conducted in the Po Delta Regional Park Emilia-Romagna, located along the North Adriatic coast of Northern Italy (ca. 44°32′–44°56′ N; 12°08′–12°16′ E; Fig 1). The Park has a total area of 54,000 ha and encompasses a great variety of habitats, including pine forests, wetlands, dunes, and dry grasslands. Consequently, several sites of community importance and special areas for biodiversity conservation are currently present in this region [https://www.parks.it/parco.delta.po.er/Epar.php (accessed on 23 April 2025)]. In addition, the northern section of this area contains the National Nature Reserve Bosco della Mesola. Based on the Köppen climate classification, the study area has a temperate continental climate, with a mean annual temperature of about 14 °C. Mean monthly temperatures vary from 3°C in January, the coldest month, to approximately 24°C in July, the warmest month. The mean total annual precipitation ranges from 600 to 700 mm, with a short period of mild summer aridity. The average annual relative humidity is approximately 70–75% [34].

We have been investigating wild orchids in the Po Delta Park since 2012. Fifteen species of native orchids are currently present in this study area [35]. In this study, we aimed to analyse the ecological niche of native orchid species by including both the highest possible number of different habitat types and ensuring the presence of sample replicates. On this basis, we selected seven orchid species: *Ophrys sphegodes* Mill.; *Anacamptis morio* (L.) R.M. Bateman, Pridgeon et M.W. Chase; *Anacamptis laxiflora* (Lam.) R.M.Bateman, Pridgeon & M.W. Chase; *Anacamptis palustris* (Jacq.) R.M.Bateman, Pridgeon & M.W. Chase; *Ophrys apifera* Huds.; *Anacamptis pyramidalis* (L.) Rich. and *Anacamptis coriophora* (L.) R.M. Bateman, Pridgeon et M.W. Chase. Specifically, O*. sphegodes*, *A. morio*, *A. pyramidalis* and *A. coriophora* inhabit dry or semi-dry grasslands that lie on sandy flat areas corresponding to the ancient dunes levelled by atmospheric agents whereas *A. laxiflora* and *A. palustris* typically occur in wet meadows [35].

### 2.2 Data collection and laboratory analyses

Field sampling was conducted from March to June 2023. We visited a total of 27 selected sites several times throughout the spring and early summer, following the species’ phenology. Eight of these sites hosted more than one of the orchid species included in the study, making a total of 35 site occurrences (Table 1). For each study site, we estimated both the spatial extent and the number of orchid individuals present. Three 1-m^2^ quadrats were established at representative points in all the sites. Within each quadrat, all vascular plant species were identified, and their abundance was visually estimated using a categorical scale: 1 = 1–10%, 2 = 11–20%, 3 = 21–30%, 4 = 31–40%, 5 = 41–50%, 6 = 51–60%, 7 = 61–70%, 8 = 71–80%, 9 = 81–90%, 10 = 91–100%. Total vegetation cover was recorded at each plot using a percentage scale. Data were collected at the peak of species flowering season following the protocol described in Braun-Blanquet (1932) [36]. Three repeated measurements of soil Volumetric Water Content (VWC) were carried out at each site using a FieldScout time domain reflectometer TDR 100 Soil Moisture Meter (Spectrum Technologies Inc, Aurora, IL, USA). Soil VWC measurements were performed several times throughout the sampling season. Three replicates of about 100 g of soil were collected at each site to a maximum depth of 5–10 cm. A Data Logger (HOBO® Pendant Temperature/Light Data Logger, Onset, Bourne, MA, USA) was deployed at each site to continuously measure soil temperature hourly.

**Table 1:** Site occurrences of species.

Species	Site occurrences
*Ophrys sphegodes*	6
*Anacamptis morio*	6
*Anacamptis laxiflora*	2
*Anacamptis palustris*	3
*Ophrys apifera*	5
*Anacamptis coriophora*	7
*Anacamptis pyramidalis*	6

The site occurrences (35) of the study species.

The soil analyses were carried out at the Laboratory of Plant Ecology (Department of Environmental and Prevention Sciences), University of Ferrara. A 10 g subsample of dry soil was extracted in a 1:2.5 (vol/vol) aqueous solution and used to determine soil pH with a pH meter [Hanna Edge, Villafranca Padovana (PD), Italy]. Electrical conductivity and salinity were determined with a Crison CM35 conductivity-meter (Hach-Lange, L’Hospitalet de Llobregat, Spain). A 10 g subsample of dry soil, sieved with a 0.125 mm mesh, was extracted in 100 mL of H_2_O, filtered with Whatman 25-mm GD/X syringe filters (pore size 0.45 μm) and colorimetrically analysed for ammonium (NH_4_^+^) and nitrate (NO_3_^−^) concentrations. The Olsen method was used to analyze phosphate (PO_4_^3−^) concentration from a dry sieved soil sample [37]. These analyses were conducted using a continuous-flow autoanalyzer (Systea Flowsys, Anagni, Italy). Specifically, ammonium was determined by the salicylate method in the presence of hypochlorite [38], nitrate by the cadmium-reduction method through a column [39], and phosphate by the molybdenum-blue method [40].

### 2.3 Data compilation and statistical analyses

To assess the ecological preferences of orchid species from both a vegetation-based and an abiotic perspective, we applied a combination of statistical approaches. Ecological differentiation analyses based on measured soil variables were performed in the R environment using the ADE-4 package [41]. To this aim, a Principal Component Analysis (PCA) was conducted to examine the overall differences among species in terms of their ecological preferences for soil properties, taking into consideration all 35 site occurrences. Subsequently, we performed an Outlying Mean Index (OMI) analysis to determine niche marginality and breadth [42]. For the analysis, we considered the 27 sites to highlight the associations between orchid species. We used two datasets: the first dataset consisted of the species presences in the sites, while the second dataset included the explanatory soil variables, i.e., soil VWC, pH, salinity, and soil nutrient contents (ammonium, nitrate, and phosphate concentrations). Species with high OMI values (niche marginality) are considered to occupy marginal niches, for being associated with environmental conditions that are relatively uncommon in the study area. Conversely, species with low OMI values inhabit non-marginal niches, reflecting their association with more typical or widespread habitat conditions. Species exhibiting low tolerance values (niche breadth) are confined to a narrow range of environmental conditions and are thus classified as specialists, whereas those with high tolerance values occupy a broader range of conditions, indicating a generalist strategy. A random permutation test with 10,000 permutations was employed to test the statistical significance of each species’ marginality.

A Multivariate Analysis of Variance (MANOVA) was performed using normalized EIVs derived from field floristic surveys for Light (L), Temperature (T), Moisture (M), Reaction (R), Salinity (S), and Nutrients (N) as dependent variables, to investigate which ecological factors most strongly influenced the occurrence of each orchid species [43]. The EIVs were normalized prior to analysis based on species composition at each site, ensuring comparability across gradients using Formula (1):


$${\text{EIV}}_{\text{normalized}}=\sum {\mathrm{\backslash nolimits}}_{i=\text{1}}^{n}\left(\frac{Ai\;x\;EIVi}{\sum Ai}\right)\;$$ (1)

where A*i* is the mean abundance of the i-th species resulting from the three vegetation surveys, EIV*i* is the corresponding Ellenberg Indicator value, and ∑Ai is the total sum of mean abundances. Statistical significance was assessed via permutation tests. To evaluate whether normalized EIVs (for Moisture, Reaction, Salinity, and Nutrients) reflected the actual environmental conditions, we tested the relationships between normalized EIVs and measured soil variables (soil VWC, pH, salinity, and inorganic nitrogen and phosphorus concentrations) using Linear Regressions. The MANOVA and the linear regressions were performed using PAST 4.16c [44].

## 3 Results

### 3.1 Soil properties and ecological niches

**Fig 2:**
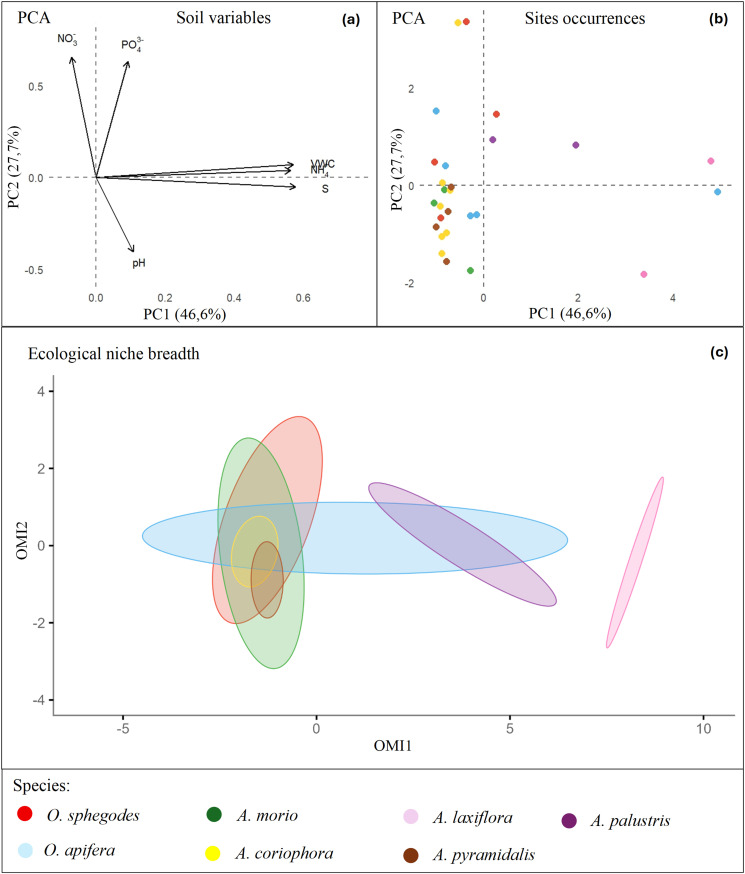
Plot of PCA and OMI analyses. **(a)**: Plot of environmental variables; **(b)**: Distribution of species along environmental gradients; **(c)**: Ecological niche breadth.

The patterns resulting from the PCA emphasized the ecological differentiation of orchid species based on measured soil variables (Fig 2a). The first two axes (PC1 and PC2) explained 48.6% and 27.3% of the dataset’s variance, respectively. PCA 1 primarily reflected a multiple gradient of soil moisture, salinity, ammonium and, to a lesser extent, soil pH. Indeed, PCA 1 separated species primarily associated with drier and less saline habitats from species associated with wetter habitats (Fig 2b). In particular, *O. apifera*, *A. pyramidalis*, and *A. coriophora* were mostly clustered along the negative side of PCA 1, corresponding to lower values of moisture and salinity. *A. laxiflora* and *A. palustris* were located on the positive side of PCA 1, indicating their association with high moisture and salinity in the soil. PCA 2 explained most of the variation in soil nutrient contents, namely soil nitrate and phosphate concentrations. *O. sphegodes* and *A. morio* had positive scores along PCA 2, denoting their association with more nutrient-rich environments. The other species had generally lower or intermediate scores on the PCA 2 axis, indicating more variation in their nutrient preference. Interestingly, *O. apifera* tended to occupy a broader range of ecological conditions, including nutrient-poor, dry habitats, as well as more saline and wet habitats.

The OMI analysis showed marginality and breadth values to vary considerably, indicating different levels of ecological specialization among species (Fig 2c; Table 2). *A. laxiflora* exhibited the highest OMI value, and thus the highest niche marginality. *A. laxiflora* also exhibited the lowest tolerance and relatively low residual tolerance, which confirms its narrow niche breath, indicating a strong association with highly distinctive environmental conditions, particularly wet and saline habitats. *A. palustris* showed the second highest marginality value, indicating a potential ecological preference for moist and nutrient-rich soils. It had a broader niche breadth, implying somewhat higher ecological plasticity compared to *A. laxiflora*. *A. pyramidalis* had intermediate values of all parameters, suggesting a moderately wide niche with limited specialization. *A. coriophora* showed low marginality, low tolerance, and highest residual tolerance, indicating that its distribution may be influenced by other environmental or biotic factors not captured in our analyses. *O. sphegodes* and *A. morio*, can be considered ecological generalists due to their low marginality values, which suggests their distribution to be centered close to average environmental conditions. *O. apifera* showed the lowest marginality value and the highest tolerance value, indicating a great capacity to adapt to a wide range of ecological conditions. Overall, the mean OMI across all species was significantly higher than expected under random distribution (OMI.mean = 3.77; *p* < 0.01), indicating that the orchid species investigated were non-randomly distributed along the environmental gradients (Fig 2c, Table 2). This supports the view that habitat filtering played an important role in shaping their distribution.

**Table 2:** OMI parameters.

Species	Inertia	Outlying Mean Index	Tolerance Index	Residual Tolerance Index
*O. sphegodes*	4.61	17.4	12.6	70.0
*A. morio*	4.40	15.8	2.7	81.5
A. laxiflora *	11.87	78.3	0.4	21.3
A. palustris *	11.87	41.6	9.9	48.5
*O. apifera*	7.45	5.5	38.6	55.9
*A. coriophora*	3.67	24.2	2.6	73.3
*A. pyramidalis*	1.50	72.2	10.0	17.8

Value of the different OMI parameters for each species. * indicates statistical significance (*p* < 0.05).

### 3.1 Ellenberg indicator values

The MANOVA revealed significant differences in the ecological factors inferred from species composition in the sampling sites (*p* < 0.01, Table 3). Specifically, *A. morio* was distinguished by notably low normalized EIV values for Salinity, Moisture, Reaction and Nutrients, which highlights its preference for dry, nutrient-poor, and slightly acidic soils. *A. laxiflora* had a strong positive association with normalized Moisture EIV value, confirming its preference for wetter environments. Similarly, *A. palustris* exhibited high positive scores for normalized Moisture and Salinity EIV values, indicating its preference for wet and saline habitats. *O. apifera* was negatively associated with normalized Light EIV value, suggesting a preference for more shaded conditions. *A. coriophora* showed a strongly negative correlation with normalized Moisture EIV value, highlighting its preference for dry soils.

**Table 3:** MANOVA of normalized Ellenberg Indicator Values.

Species	L (± SE)	T (± SE)	S (± SE)	M (± SE)	R (± SE)	N (± SE)
*O. sphegodes*	−0.08 ± 0.20	0.08 ± 0.31	−0.16 ± 0.13	−0.35 ± 0.23	0.05 ± 0.22	−0.01 ± 0.18
*A. morio*	0.11 ± 0.20	−0.11 ± 0.31	−0.29 ± 0.13*	−0.66 ± 0.23**	−0.54 ± 0.22*	−0.63 ± 0.18**
*A. laxiflora*	0.38 ± 0.32	−0.32 ± 0.50	0.50 ± 0.20*	0.03 ± 0.37	0.23 ± 0.35	0.18 ± 0.30
*A. palustris*	0.03 ± 0.27	−0.64 ± 0.42	0.36 ± 0.17*	1.61 ± 0.31***	0.27 ± 0.29	0.49 ± 0.25
*O. apifera*	−0.49 ± 0.22*	0.53 ± 0.34	−0.01 ± 0.14	0.45 ± 0.25	0.13 ± 0.23	0.29 ± 0.20
*A. pyramidalis*	−0.20 ± 0.20	0.37 ± 0.31	−0.24 ± 0.13	−0.39 ± 0.23	−0.03 ± 0.22	−0.02 ± 0.18

Mean (±1 SE) values of normalized Ellenberg Indicator Values (EIVs) for Light (L), Temperature (T), Salinity (S), Moisture (M), Reaction (R), Nutrient (N) by Multivariate Analysis of Variance (MANOVA). *p*-values are indicating as follows: *p < 0.05; **p < 0.01; ***p < 0.001.

**Fig 3:**
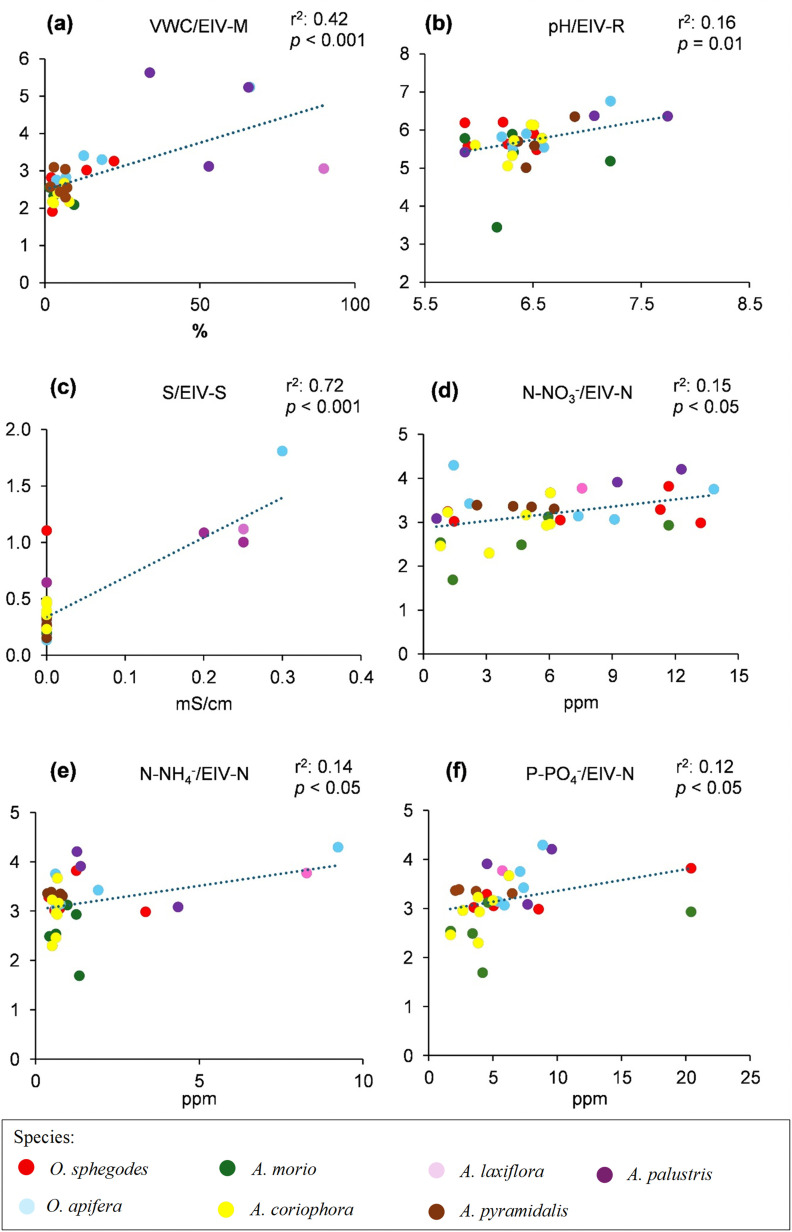
Linear regression between normalized Ellenberg Indicator Values (EIV) and field measurements. **(a)**: EIV-Moisture/Volumetric Water Content (VWC); **(b)**: EIV-Reaction/pH; **(c)**: EIV-Salinity/Salinity; **(d)**: EIV-Nutrient/Nitrate concentration (NO_3_^-^); **(e)**: EIV-Nutrient/Ammonium concentration (NH_4_^+^); **(f)**: EIV-Nutrient/Phosphate concentration (PO_4_^3-^). r-squared and *p*-values are indicated within each panel.

Linear regression analyses showed varying levels of agreement between normalized EIVs and measured soil variables (Fig 3a-f). The strongest relationship was found for the Salinity index (Fig 3c), indicating that species composition effectively captured soil salinity variation across sites. Soil VWC also showed a significant correlation with the Moisture index, confirming the reliability of EIV-M in reflecting soil moisture gradients (Fig 3a). Weaker relationships were observed between the Reaction index and soil pH (Fig 3b), as well as between the Nutrient index and the concentrations of nitrate, ammonium and phosphate (Fig 3d-f). Although statistically significant, the lower r^2^ values associated with nutrient variables suggest a weaker predictive power of EIV-N for capturing local soil fertility conditions.

## 4 Discussion

Our study provided consistent evidence that soil properties had a pivotal role in shaping the distribution of orchids. The PCA 1 axis separated *A. coriophora* and *A. pyramidalis*, both associated with drier and less saline soil conditions on one side, from *A. laxiflora* and *A. palustris*, which both preferred more humid and saline habitats on the other side. Soil moisture is generally considered as a major ecological factor affecting the distribution and population density of orchid species, even at the microsite scale [45,46]. Beyond its direct influence on plant growth, soil moisture significantly affects nutrient cycling, soil chemistry and thermal properties of the soil [47,48]. The PCA revealed that most of the species were found in nutrient-poor habitats, especially *A. coriophora* and *A. pyramidalis*. It is well known that many terrestrial orchid species prefer nutrient-poor soils where they depend on fungal associates for acquiring nutrients [49,50]. Infertile conditions may themselves promote both the establishment and the long-term stability of these mutualistic relationships and hence the creation of a balanced ecological niche. In such conditions, competition with fast-growing plants adapted to nutrient-rich conditions is reduced, while orchids benefit from their efficient capability of acquiring nutrients by their symbionts [51]. On the other hand, *O. sphegodes* and *A. morio* were generally found at sites with higher nitrate and phosphate contents in the soil, probably due to increased nutrient input from adjacent crops. Interestingly, high phosphorus availability has been found to enhance the diversity of mycorrhizal fungal communities associated with two *Bipinnula* species in Chile [52]. Overall, this suggests that soil nutrient availability not only affects the distribution of wild orchid species but also modulates their fungal associations, potentially promoting specialization in nutrient-enriched habitats. Both the PCA and the OMI analyses showed that *O. apifera* had the lowest marginality and the highest tolerance. Previous studies found that *O. apifera* can colonize different types of semi-natural habitats, including anthropogenic environments, primarily on xerophilous or mesoxerophilous soils [53–55]. In our study area, *O. apifera* proved to be adapted to moderately wet conditions. *A. morio* and *O. sphegodes* also presented low marginality values, indicating their ecological flexibility and adaptation to habitats close to the regional average. Generalistic strategies are often associated with broader ecological tolerance, higher phenotypic plasticity, varied resource usage, and stronger resilience to environmental disturbances [56]. However, such adaptability can sometimes mask subtle fine-scale dependency on microhabitat features or mycorrhizal associations, which require further investigation. On the other hand, specialistic species tend to occupy narrow, often extreme ecological niches [56], in fragmented micro-habitats sensitive to disturbance, which makes specialistic plants particularly vulnerable. Among the species investigated, *A. laxiflora* exemplifies this trend since it exhibited highest marginality and lowest tolerance, which indicates a narrow ecological niche, primarily defined by high moisture and salinity. Similarly, *A. palustris* showed substantial marginality, although combined with slightly broader tolerance, suggesting considerable ecological plasticity within its preferred wet environments. On the opposite end of the gradient, *A. pyramidalis* was typically found in drier, infertile soils with a preference for open, xeric grasslands. Although seemingly more resilient, these dry habitats are equally subject to degradation through land abandonment or encroachment of more competitive species [57]. These findings are in line with the general ecological principle that most highly specialized species are found at the extreme ends of environmental gradients [58]. Therefore, many orchid specialists inhabit either wet habitats, such as fens or wet meadows, or dry and warm environments like calcareous grasslands [47]. From a conservation perspective, ecologically marginal species seem to be generally more affected by ongoing events as climate change, genetic drift and human influence, leading to disturbances in their survival [59]. As a result, they are more likely to experience range contractions or local extinctions under changing conditions compared to generalist species, although some variation in the occurrence and rarity of orchid species may be found due to the biogeographical context [60,61]. A recent study [62] highlighted that the geographic ranges of European orchids are shaped more by species-specific biological characteristics and abiotic conditions rather than by evolutionary history. As shown by our results and previous studies [4,63], soil properties play a major role in affecting orchid distribution. Therefore, conservation efforts should focus on preserving a broad range of environmental conditions across landscapes [64]. Our results stress the importance of maintaining environmental heterogeneity to sustain orchid diversity, since it allows species to respond and adapt to environmental changes as an urgent need in the context of climate change and habitat degradation [65]. Local studies addressed to orchid species with narrow ecological ranges can offer valuable insight for regional planning and habitat restoration, especially in Mediterranean ecosystems facing increasing anthropogenic pressure [66,67]. This perspective is particularly relevant in the context of the Po Delta Regional Park, a complex mosaic of protected and non-protected areas heavily impacted by growing urbanization and agricultural expansion, where the actual environmental protection and policy are not effective in the conservation of wild orchid populations [33]. Under such conditions, the preservation of microhabitats that support niche-specific conditions, such as saline depressions, wet meadows, and calcareous dry grasslands, becomes even more critical. Fragmentation, land-use change, and alien plant invasion can disproportionately affect ecologically marginal species, which are often confined to narrow habitat patches within this landscape. We used EIVs to test whether floristic composition reflects the same environmental gradients revealed by soil measurements. The MANOVA confirmed that the strongest interspecific differences were observed along the moisture and salinity gradients, with *A. palustris* and *A. laxiflora* standing out for their affinity for wet saline habitats, mirroring the environmental gradients detected in the PCA.

Linear regression analyses revealed different degrees of correspondence between normalized EIVs and soil properties measured in the field. Weaker correlations were observed for the reaction index (EIV-R) and especially for the nutrient index (EIV-N), indicating a lower effectiveness of these indicators in predicting soil pH and fertility, which supports the results of previous studies. For example, Schaffers and Sýkora [68] found weak correlation between EIV-N and soil nutrient concentrations in 14 different Dutch roadside plant communities, probably due to the high temporal variability of soil nutrient contents and their complex interaction with community structure. The low correlation between EIV-N and the concentrations of nitrate, ammonium and phosphate in the soil could also reflect indirect effects of unmeasured edaphic factors, such as soil retention capacity, or interspecific competition for resources which may influence vegetation composition in a non-linear manner [69]. Moreover, soil nutrient contents can undergo strong seasonal fluctuations related to microenvironmental heterogeneity [70,71]. The reaction index (EIV-R) also had weak correlation with soil pH, probably because most of the orchid species investigated usually occur on soils with a narrow pH range from slightly acidic to slightly alkaline [72]. In contrast, the strongest correlation was found between the Salinity index and soil salinity, followed by a high correlation between the Moisture index and soil Volumetric Water Content. This suggests that floristic composition was an effective indicator of soil salinity and moisture variability in the habitats investigated. In summary, our results support both the reliability of EIVs as ecological proxies in vegetation-based analyses, with better performance for temporally stable variables (e.g., moisture, salinity) and weaker performance for dynamic variables such as soil nutrients [73,74].

## 5 Conclusions

Our study revealed distinct environmental gradients shaping wild orchid species distribution and highlighted differences in their ecological strategies, distinguishing generalist orchids (*O. apifera*) from specialists with narrow ecological ranges (*A*. *laxiflora*). These findings highlight that conservation strategies must account for habitat specialization and environmental heterogeneity, particularly where specialist species occur at the extremes of ecological gradients. Therefore, integrating fine-scale ecological data into regional conservation planning is essential to ensure that the full range of orchid diversity is protected in the long term, especially within human-dominated ecosystems like the Po Delta.
